# The Current Description and Future Need for Multidisciplinary PCOS Clinics

**DOI:** 10.3390/jcm7110395

**Published:** 2018-10-28

**Authors:** Wendy M. Wolf, Rachel A. Wattick, Olivia N. Kinkade, Melissa D. Olfert

**Affiliations:** 1 Physician Assistant and Registered Dietitian at Apex Family Medicine Denver, Denver, CO 80209, USA; wendywolf02@gmail.com; 2 Department of Human Nutrition and Foods, West Virginia University, Morgantown, WV 26506, USA; rawattick@mix.wvu.edu (R.A.W.); onk0001@mix.wvu.edu (O.N.K.)

**Keywords:** PCOS, multidisciplinary, comprehensive treatment, PCOS Clinics

## Abstract

Polycystic Ovarian Syndrome (PCOS), with common symptoms of irregular menstrual cycles, ovarian cysts, and hirsutism, is thought to be the most common endocrine disorder found in women, and use of multidisciplinary teams has been shown to be effective. The purpose of this review is to determine the future need for specialized, comprehensive, multidisciplinary treatment for PCOS and the current description and efficacy of existing multidisciplinary clinics. The literature was searched using PubMed, CINAHL, PsycINFO, Medline, and the Cochrane Library. Keywords included treatment efficacy, polycystic ovary syndrome, treatment and collaboration. Results showed that while an increasing number of studies continue to come out expressing the need for multidisciplinary approaches to and clinics for the treatment of PCOS, there is still a large gap in the literature documenting actual multidisciplinary PCOS treatment facilities. The limited literature documenting the efficacy of multidisciplinary PCOS clinic have demonstrated increased weight loss, high patient satisfaction, and high retention compared to single-care providers. Data showed that these teams are most commonly made up of a combination of endocrinologists, psychologists, dietitians, gynecologists, and endocrine-specialized nurses. Data showed that there is a high degree of variability and rates of diagnosis between types of single-care providers, such as: endocrinology, dermatology, gynecology, and fertility. Individuals with PCOS are in need for specialized, individualized, and focused care from a diverse team of healthcare providers to treat PCOS comprehensively.

## 1. Introduction

Polycystic ovarian syndrome (PCOS) is thought to be the most common endocrine disorder found in women [1,2]. PCOS has a variety of phenotypes; therefore, it presents a broad spectrum of clinical symptoms and risk factors. Common symptoms include irregular menstrual cycles, ovarian cysts, and hirsutism [2]. PCOS impacts women of all races and ethnicities who are of childbearing age. PCOS is associated with a significant increase in risk factors such as cardiovascular disease, type 2 diabetes, and infertility [3]. The etiology of PCOS is not completely understood, although genetic and lifestyle factors are known to influence the etiology and insulin resistance plays a key role in the pathogenesis of PCOS [3]. Insulin resistance is thought to play a central role in the etiology of PCOS and is present in 50–90% of women with PCOS (depending on diagnostic criteria used), which is significantly worse than age- and BMI (body mass index) -matched control women [4]. Data estimates that 38–88% of women with PCOS are overweight or obese across the world, with an increased rate in the United States to mirror the higher obesity rates in the non-PCOS population [4]. Insulin resistance does present in individuals with lean PCOS, as well as overweight and obese women. Common treatments for insulin resistance include Metformin and weight reduction/lifestyle interventions. Another characteristic feature of PCOS is hyperandrogenism, which refers to elevated male hormones (androgens), such as testosterone. Hyperandrogenemia can be diagnosed clinically through the presence of acne, hirsutism (unwanted hair growth around the face, chest, or trunk), or alopecia (male-pattern baldness or the thinning of hair). It can also be diagnosed biochemically through a blood test. In a large study of over 1000 women with androgen excess, 659 presented with hirsutism and 78.4% of the hirsute women were diagnosed with PCOS under the 1990 NIH criteria [5]. Common treatment of hyperandrogenic symptoms include spironolactone or finasteride. PCOS is often associated with infertility, which is present in an estimated 40% of women diagnosed with PCOS [6]. The root of infertility in these women is likely from reoccurring menstrual disturbances, which is often presented as oligomenorrhea (with 85–90% of women with PCOS), amenorrhea (present in 30–40% of women with PCOS), or abnormally long or erratic menstrual patterns [6]. It is also important to mention that up to 30% of women diagnosed with PCOS have normal menstrual cycles, which emphasizes the high degree of heterogeneity in this condition. Mental health outcomes are also a concern when examining a multifaceted condition with dermatological symptoms, weight gain, fertility issues, as well as a variety of risk factors. Shakerardekani et al. conducted a cross-sectional, multi-centric study in Iran on 100 women with PCOS and found that 45% presented with depression and 30% were considered for possible cases of other mental disorders [7]. Another study by Coffey et al. in the UK found that PCOS had a negative impact on health-related quality of life, even when compared with a variety of other health conditions with a relatively small sample size using a reliable and valid evaluation tool [8]. 

Given the heterogeneity of its symptoms and its constantly evolving (and debated) diagnostic criteria, the prevalence of PCOS can be difficult to pinpoint. The prevalence of PCOS is commonly thought to vary between 5% and 10% depending on diagnostic criteria and sample population [1,2,3]. It has been previously stated that because PCOS is a clinical syndrome, and there are no criteria that are fully sufficient for diagnosis [4]. There are three different sets of diagnostic criteria (see Table 1) that are used in the field, set by National Institutes of Health’s (NIH) international conference on PCOS in 1990, the European Society of Human Reproduction and Embryology and the American Society for Reproductive Medicine (ESHRE/ASRM) in 2003 (referred to as the Rotterdam criteria), and the Androgen Excess Society & PCOS Society (AE-PCOS) in 2006 [3,9,10,11]. Each set of criteria has slightly different clinical, biological, and image-based findings to determine the presence or absence of PCOS [3]. The Rotterdam 2003 Criteria were developed in response to a need for broader diagnostic criteria [1]. To be diagnosed with PCOS under the Rotterdam criteria (ROT), the individual must exhibit symptoms in two of the three categories, which include oligo/anovulation, hyperandrogenism, and the presence of polycystic ovaries [3]. The most recent criteria, published by the AE-PCOS in 2006, tightened the parameters to include all three symptoms used in the Rotterdam criteria in an effort to give an evidence-based definition to PCOS [3,9]. All three diagnostic criteria sets have specific exclusion criteria that differ. The diagnostic criteria are constantly evolving and are considered to be one of the most debated topics in the field of endocrinology [12], making the prevalence of PCOS difficult to determine with consistency. It has been previously stated that because PCOS is a clinical syndrome, there are no criteria that are fully sufficient for diagnosis [12]. 

Based on the NIH diagnostic criteria, there is a similar prevalence of PCOS of between 6% and 9% documented across the United States, the United Kingdom, Spain, Greece, Australia, and Mexico [13]. This information suggests that there are no racial or ethnic influences on the prevalence of PCOS. Due to the lack of comparability among the studies, biased group selections, and small sample sizes, it is recommended that further research be conducted before this generalized statement is accepted. There are multiple hypothesized reasons for the lack of understanding of the risk and diagnosis of PCOS and one main reason could be the conflicting diagnostic criteria. The different components of the diagnostic criteria cause alterations in the prevalence, as well. National prevalence rates have been reported at as low as 1.6% using a combination of all three criteria [1] and as high as 6.6% using 1990 NIH criteria in similar American populations [13]. There is limited literature that exists, but similar prevalence rates between Whites of European decent, African-American, and Mexican women have been noted [14]. 

The main goal of this review is to determine the need for multidisciplinary PCOS clinics based on their treatment contribution and assess the current literature on existing multidisciplinary PCOS clinics.

## 2. Material and Methods

The goal of this review was to assess the current literature on existing multidisciplinary PCOS care or data to support the need for a variety of specialist in hopes of promoting future research and advancements in clinical practice for women with PCOS. A review of published peer-reviewed literature was conducted by searching the following the databases: CINAHL, PsycINFO, Medline, and the Cochrane Library. The keywords searched were PCOS, polycystic ovary syndrome, polycystic ovarian syndrome, multidisciplinary, clinic, and treatment. Additional articles were suggested by the authors and clinicians involved in this review. The most recent search was conducted on 15 October 2018. Studies were first selected based on title and abstract. A full text assessment was then utilized to ensure relevance. Studies were included if the title and abstract focused on the efficacy or description of multidisciplinary PCOS clinic. For this review, the term ‘multidisciplinary’ will refer to at least two practitioners from different specialties who are providing care or treatment individuals with PCOS. 

## 3. Results

### 3.1. Significance of the Problem

PCOS is a multifaceted and complex syndrome that requires care from multiple providers to fully treat the full spectrum of the condition. It can be difficult to treat due to its heterogeneity between patients, which therefore requires specialized, individualized and focused care. PCOS is most often diagnosed in adolescents struggling with menstrual issues or women with infertility; combine those issues with hirsutism, high insulin levels, weight-gain, and acne [1], and this leads to a vulnerable and frustrated population. There are many aspects involved in evaluating and treating PCOS patients, including: regulating the menstrual cycle, addressing metabolic abnormalities, normalizing nutrition (i.e., weight loss if overweight), psychological treatment for poor self-image, depression, and anxiety, and addressing concerns such as infertility and increased risk of comorbidities [15,16]. A multidisciplinary treatment approach to PCOS is thought to be an effective strategy that will enable the coordination of care and also serve as an innovative platform for research across the full spectrum of PCOS [17].

Another reason to support the need for multidisciplinary PCOS clinics is the high rate of PCOS patients who remain undiagnosed when only seeing one specialist, as demonstrated in a study by Broder-Fingert et al. [18]. Data was collected on an inner-city clinic by a retrospective chart review in a hospital-based pediatric clinic in New York City. Data was analyzed from 60 female patients between the age of 13 and 19 with a primary ICD-9 (International Classification of Diseases) diagnosis of ovarian dysfunction, menstrual irregularity, or hirsutism who were randomly selected. The primary outcome of this study looked at the rates of assessment for the diagnostic criteria of PCOS and selected co-morbidities in an inner-city pediatric clinic. Only 25% of the patients in the study with suspected PCOS (according to any of the three components of the Rotterdam criteria: menstrual irregularity, hyperandrogenism, and ultrasonographic features of PCOS) had even been evaluated for PCOS [18]. Only 2 patients (3.33%) had been evaluated for common co-morbidities associated with PCOS [8]. A full evaluation for PCOS at this clinic included: report of menstrual irregularities, clinical signs of hirsutism, ovarian ultrasound, fasting glucose, fasting insulin, lipid profile (HDL-high-density lipopotein, LDL-low-sensity lipoprotein and triglycerides), testosterone, and FSH-follicle-stimulating hormone to LH-luteinizing hormone ratio. Twenty-eight out of 60 patients presented with menstrual irregularity in addition to one of the following signs: obesity, hirsutism, and/or acne [18]. Of those 28 patients, only 15 (54%) were evaluated for PCOS (according to the Rotterdam Criteria) and only 7% were evaluated for co-morbidities [18]. The lack of specified PCOS evaluation in patients with one or more symptoms demonstrates that PCOS is under-evaluated and under-diagnosed in this pediatric population and most likely throughout the US. This study’s strengths included its exhaustive evaluation measures to diagnose PCOS, and the fact that it is the first study to assess the rate of undiagnosed PCOS cases in an inner-city population. Due to the reliance on a retrospective chart review, there is a possibility of improper medical chart reporting, which could alter the rate of existing diagnosis. 

Sivayoganathan et al. [19] showed that the percent of patients that go undiagnosed with PCOS might vary greatly depending on the type of clinic. The data for this study was collected on 70 women using a prospective cross-sectional observational study at four different clinics at Leeds General Infirmary in the United Kingdom. Participants were all assessed for PCOS with a full endocrine and metabolic profile as well as an ultrasound. In this study, 65% attending the dermatology clinic, 38% attending the endocrinology clinic, 25% attending the gynecology clinic, and 15% attending the fertility clinic were confirmed by this study to have PCOS without a pre-existing clinic [19]. The difference between the rates of existing diagnosis was shown to be significant (*p* = 0.0088) [19]. Even though all participants experienced menstrual problems, there was a significant difference (*p* < 0.0234) with menstrual patterns and the frequency distribution of related symptoms between types of clinics [19]. This study also found that four out of six participants who were found to have diabetes were diagnosed through this study, indicating a poor rate of treatment and diagnosis [19]. Most women were receiving an oral-glucose test for the first time. This was the first study to compare four different clinics that often treat the symptoms of PCOS, and it also used an exhaustive evaluation of PCOS. This study may have been limited by a relatively small number of participants but significant results were still found.

Additionally, research has continually shown that a reduction in weight as low as 5% can significantly improve a variety of issues ranging from hyperandrogenism, to insulin resistance and infertility [20]. A 2017 study done by Faghfoori et al. asserts that if more women can have access to resources such as established, multidisciplinary PCOS treatment clinics where they may be promptly and more properly diagnosed, they may sooner reap the profound multi-domain benefits of even a small change in health behaviors [21].

### 3.2. Efficacy of Multidisciplinary PCOS Clinics

Very few studies have been done to assess current multidisciplinary treatment for PCOS. It is important to understand the clinical variability that is seen between treatment providers, success in existing clinics, and additional research promoting the use of a multidisciplinary treatment team. A cross-sectional study to assess practice heterogeneity by Bonny et al. [22] conducted an anonymous Internet survey that yielded 127 responses from North American Society of Pediatric Adolescent Gynecology (NASPAG) members. When the respondents were asked to select their expertise (more than one could be selected) 64% selected gynecology, 43% selected adolescent medicine, 13% selected reproductive endocrinology, 6% selected general pediatrics, and 4% selected endocrinology [22]. The most common first-line treatment therapies included the prescription of oral contraceptives followed by diet modification and exercise with 98% and 90% of respondents, respectively [12]. While 65% of clinicians would not make a diagnosis for PCOS within the first two years after menarche, 35% of respondents would [22]. Only 60% of respondents noted that they look at blood glucose levels at an initial PCOS evaluation [22]. Thyroid stimulating hormone (TSH) was the most common test completed at an initial PCOS evaluation at just below 90% [22]. There was a high degree of variability between the hormonal and metabolic evaluation. The high degree of variability in treatment is evidenced by the fact that there was not one test used to diagnose PCOS that was ordered by all clinicians. Even though this survey targeted the experts in the field who have an interest in PCOS, there was a considerable amount of heterogeneity within the first diagnostic testing that was normally completed. This study was limited due to it being a pilot study that was designed to assess descriptive statistics only, but it was the first study to address the input across a variety of providers on the direct treatment for PCOS. A breaking study by Teede et al. continues to highlight that failure to engage multidisciplinary practices is a primary factor in lack of success concerning treatment of PCOS, and that this gap must be bridged going forward as more stringent guidelines for treatment are established [20].

Currently, there are only two multidisciplinary PCOS treatment facilities that have published research regarding the outcomes of their clinics. The first multidisciplinary clinic to examine the benefits of such treatment is one of the most well-known multidisciplinary PCOS clinics in the United States, which is at the American Family Children’s Hospital in Madison, Wisconsin. This clinic has been in existence since 2005. This clinic’s team consists of two pediatric endocrinologists, a reproductive endocrinologist, an endocrine nurse, a health psychologist, a dietitian, and a pediatric gynecologist [15,16]. The following two studies were based on data collected during the first 33 months of operation (March 2005–December 2008) of this clinic. The goal of this study was to characterize patients referred to the adolescent PCOS clinic at the American Family Children’s Hospital by conducting a chart review of all patients (*n* = 70) seen in the first 33 months. Bekx et al. collected data on initial presentation, age, body mass index (BMI), menstrual pattern, clinical and laboratory features of androgen excess, insulin resistance, and dyslipidemia. The average age of the patients at the time of referral was 16.2 years old but ranged from 11 to 22 years old [15]. Eighty-four percent of patients had a BMI above the 85th percentile and 70% had a BMI greater than the 95th percentile [15]. Only 16% of the patients had a normal BMI of less than the 85th percentile [15]. They saw a great amount of variance in menstrual pattern and that ranged from primary amenorrhea to regular cycles. Over 50% of patients showed signs of hirsutism [15]. Only three cases of type 2 diabetes were confirmed, with two being pre-diagnosed and one diagnosis being given through an oral glucose tolerance test (OGTT) [15]. More than 50% of patients were thought to have insulin resistance that was demonstrated by elevated fasting insulin levels or a fasting glucose-to-insulin ratio of less than 4.5 [15]. Twenty-four percent of patients had elevated triglyceride levels above 150 mg/dL, and 54% had low HDL levels below 50 mg/dL [15]. Due to the retrospective design of the chart review for data collection, limitations may result from coding discrepancies. Also, because the patients were all at different stages of evaluation and treatment it could cause a variance or skew in data collected. This study did not use confirmatory testing to ensure the findings of various diagnostic measure due to the lack of financial justification. Missing data was a slight limitation, because not all labs that were ordered were actually collected, which is consistent with realistic expectations in clinical settings.

Geier et al. focused on assessing specific types of providers seen by patients, weight loss and retention rates at the same clinic. The data for this study was collected by a retrospective chart review that evaluated 140 adolescent females who had been seen at the American Family Children’s Hospital PCOS Clinic between March of 2005 and December of 2008. The average age of patients at their initial visit to the clinic is 15.9 [16]. The goal of this clinic is to have all patients see each of the five providers at the initial visit. Only 41% saw all five providers during the initial visit. All patients at this clinic saw a pediatric endocrinologist and the endocrine nurse at their first visit while an additional 60.9% saw a health psychologist, 75.5% saw a dietitian, and 70.9% saw a gynecologist [16]. Geier et al. found that nearly 70% of patients succeeded in short-term weight stabilization and 57% established weight loss [16]. These patients had an average initial BMI of 34.7 (kg/m^2^) and 76% had an initial BMI greater than the 95th percentile [16]. This study also found that 71% of patients returned for a follow-up visit with an average time of 4.5 months in between visits [16]. A retention rate this high may signify that patients are generally satisfied with the treatment they are receiving. These high rates of success may be at least partially attributed to self-selection to seek weight-loss treatment indicating a pre-existing motivation. These results might not be as pronounced in a PCOS population who have not yet reached that stage of change or older adult populations. 

The second clinic that has assessed the outcomes of multidisciplinary PCOS treatment is the Royal Berkshire Hospital in Reading, Berkshire, United Kingdom. The first study by Ghosh et al. was a two-year audit that was conducted on patients attending the multidisciplinary PCOS clinic at the Royal Berkshire Hospital from its opening in 2002 for two years until 2004 [23]. They analyzed baseline characteristics, adequacy of investigations, efficacy of treatment, and patient satisfaction. The main complaints of the 127 women who attended this clinic were weight gain, hirsutism and oligomenorrhoea [17]. These women had a median age of 30 years and a median BMI of 32 (kg/m^2^) [17]. The majority (55%) of their patients were offered Metformin for treatment but 12 (17%) discontinued use because of undesirable side effects [17]. Patients using Metformin did see a significantly greater amount of weight loss (*p* < 0.0001), with a median loss of 8 kg, than patients who did not use Metformin [17]. Fifty percent of Metformin users with hirsutism saw an improvement in Ferriman Gallwey (F-G) score, but it was less effective in those with very high initial F-G scores [17]. Metformin also significantly improved menstrual cyclicity from a median of 20 weeks to 5 weeks (*p* < 0.001) [17]. Eight of the 17 women taking Metformin and attempting to get pregnant had successful pregnancies in the two-year time audit [17]. Greater improvements to hirsutism levels were seen with the 23 women who were seeking treatment using Spironolactone. The median F-G score fell from 19 to 11 over an 8-month time frame [17]. It is thought that there could be a higher patient satisfaction related to multidisciplinary clinics than with individual health care professionals. A study conducted at the Royal Berkshire Hospital in the UK showed that 42 out of 43 patients who completed a patient satisfaction survey reported that a multidisciplinary, dedicated PCOS clinic was “useful and that they were very happy with the results” [21]. The patient satisfaction survey should be administered to a greater number of patients to more adequately represent that clinic population. The questions and response options should also be more specific to provide for a better, more well-rounded picture.

In 2007, Eldridge et al. [24] conducted an audit on a nurse-led PCOS weight management clinic at the Royal Berkshire Hospital in the United Kingdom. This clinic had participants meeting once a month for individual appointments. The clinic focused on physical activity levels, use of food diaries, medications, changes in menstruation, and weight loss data. They found that there were 61 women who had attended the clinic for at least two months. They had a mean starting BMI of 37.8. Out of the 37 women who attended for at least three months, 32 had lost weight with a mean loss of 2.88 kg. Five of those 32 women lost 5% of their total body weight, 2 lost 7.5%, and three participants lost 10%. The average weight loss increased to 4.94 kg when women had attended for at least six months. Of the women who lost at least 5% of their body weight, 71% had kept a food diary, 86% had increased their exercise levels, 86% took Metformin, and 7% took Orlistat [24]. Because participation was voluntary, these individuals were self-motivated enough to enroll, which could positively affect their results.

While lack of established multidisciplinary PCOS treatment clinics still remains a pressing issue, continued studies are coming out to support the conceptual merit of a multi-practice approach and the need for more of these clinics following successful research data. Jiskoot et al. and Zaremobini et al. both cite data that show the effectiveness of employing mixed methods in the treatment of PCOS, placing a priority on combining cognitive behavior therapy with lifestyle modifications, including energy-restricted diets and exercise with data showing that lifestyle modifications alone provide significantly fewer effective results. Following these recommendations, both studies will strive to improve the quality of life in diagnosed PCOS patients through multidisciplinary modalities [25,26]. 

With the strong association between obesity, abdominal obesity, insulin resistance and features of PCOS, weight loss is supported by the Androgen Excess PCOS Society (AE-PCOS) as part of lifestyle intervention as the primary treatment option for overweight and obese women with PCOS [27]. As previously highlighted, several studies have shown than even a small amount of weight loss, accounting to 5% of one’s body weight, can reduce the severity of the symptoms for PCOS [27]. A review by Moran et al. was conducted to determine the most effective way for women to lose weight. It was determined that achieving weight loss or even maintaining one’s weight by preventing weight gain is best done with assistance from a multidisciplinary team, to address not only lifestyle modification needs, but the high prevalence of depressive and anxiety symptoms in this population [28,26]. This multidisciplinary team should focus on lifestyle management that includes dietary, exercise, and behavioral therapy [28]. Behavioral therapy should focus on psychosocial stress while openly discussing the practical and physiological barriers associated with weight management or weight loss [29]. Current evidence is only strengthening these notions, with a 2018 study revealing that when combined with lifestyle modifications such as exercise and improved nutrition, cognitive-behavioral therapy (CBT) resulted in superior weight loss and improvement of quality of life to a significant degree when compared to populations that pursued lifestyle modification alone (3.2 kg weight loss vs. 1.8 kg, improvement in quality of life by 3.7 points vs. 1.2 points) [30]. Contrast studies done across all age groups of women with PCOS, from adolescent to peri-natal women, support evidence for CBT being significantly more effective in aiding weight loss than lifestyle modifications alone [31,32]. This is not meant to undermine the effects of lifestyle modification, however, as Thomson et al. showed that diet and exercise alone are capable of significantly increasing health confidence, psychological outlook, and even social interaction [33].

Another review article by Moran et al. sought to compare the effect of different diet compositions on a variety of outcomes related to PCOS using the findings of six articles [34]. This review showed that there were only slight differences between the diets that have been tested with PCOS patients. They saw a slightly greater weight loss with a monounsaturated fat-enriched diet; improved menstrual regularity for a low-glycemic index diet; increased free androgen index diet; improved quality of life for a low-glycemic index diet; and improved depression and self-esteem for a high-protein diet [34]. The findings of this compilation of research were inconclusive to support significant differences between any of the diet examined. The findings were conclusive in saying that any diet aimed at reducing weight could lead to clinical benefits for those with PCOS. This indicates that weight loss is more important than dietary composition when it comes to PCOS symptom management. In terms of dietary supplementation; however, a recent study done by Jamilian et al. revealed that daily provision of 1000 mg of omega-3 fatty acids and 400 IU of vitamin E to women with PCOS actually reduced depressive symptoms and inflammation levels, while improving gene expression abilities in the intervention group [35]. While it was not reported if these supplements did not directly affect weight loss itself, decreases in depression and inflammation may be helpful to encourage further weight loss behaviors, indicating that appropriate supplementation in conjunction with a weight-loss diet might improve overall levels of success [36]. Given the importance of healthy weight management demonstrated in this review, this emphasizes the need for registered dietitians to play an integral role in the management of PCOS. 

A newer, and very important, area of study is the effect of PCOS on sexual dysfunction, and subsequent depression. A pilot study by Battaglia et al. [37] investigated whether PCOS patients had an increased incidence of depression and sexual dysfunction compared to non-PCOS patients, as well as clitoral volume and vascularization change with the different androgen levels of PCOS patients. They compared 25 lean PCOS women with 18 healthy non-hirsute controls through ultrasonographic, Doppler, hormonal, and biochemical evaluations. They also administered the Italian McCoy Female Questionnaire (MFSQ) to measure female identity and evaluated depressive symptoms using the Beck Depression Inventory (BDI). They found elevated androgen levels in the PCOS patients compared to controls, but no significant differences in clitoral body volume, MFSQ, or BDI scores. Although this study did not find major differences in sexual dysfunction or depression, more research needs to be done in this area. 

## 4. Conclusions

There is limited evidence directly related to multidisciplinary PCOS clinics and the efficacy of their treatment. It is well accepted that PCOS is multifaceted and has a high degree of heterogeneity among individuals with the syndrome. When treating a patient with PCOS it is important to focus on treating the patient’s initial needs while decreasing the risk of long-term risk factors. Symptoms will be better treated if the patient is treated by a variety of specialists all working together. When individuals are exposed to multiple providers it is less likely that a PCOS diagnosis will go unseen. The sooner PCOS is identified and treatment is initiated the quality of life and prognosis of the syndrome will improve. The perceived benefits of multidisciplinary clinics globally include improved patient satisfaction, greater weight loss, improved body image, and better management of PCOS from a holistic standpoint.

Further research is needed to assess additional existing multidisciplinary clinics to determine patient satisfaction and treatment prognosis compared to those seeking treatment from only one provider. More research is also warranted to gain a better understanding of evidenced-based guidelines for treatment of PCOS, especially when considering dietary recommendations. 

## Figures and Tables

**Table 1:** Diagnostic criteria for PCOS.

NIH 1990	Rotterdam 2003	AE-PCOS Society 2006
• Hyperandrogenism • Chronic Anovulation ---Both criteria needed	• Hyperandrogenism • Oligo-and/or anovulation • Polycystic ovaries ---2 of 3 criteria needed	• Hyperandrogenism • Ovarian dysfunction ---Both criteria needed
First developed and most commonly used criteria today	Formulated to expand on NIH diagnostic definition	Formulated to provide an evidence-based definition
